# An Account of an Epidemic Sore Throat Which Appeared at Chesham, in Buckinghamshire, in the Year 1788

**Published:** 1789

**Authors:** Henry Rumsey

**Affiliations:** Surgeon at Chesham, and Member of the Corporation of Surgeons of London


### LONDON MEDICAL JOURNAL.

I. *An Account of an epidemic Sore Throat which appeared at Chesham, in Buckinghamshire, in the Year* 1788. *Communicated in a Letter to Dr.* Simmons *by Mr.* Henry Rumsey, *jun. Surgeon at Chesham, and Member of the Corporation of Surgeons of London.*

THE disease I am going to describe appeared in Chesham in Buckinghamshire, and the neighbourhood, in April, 1788, and continued to prevail more or less till the month of November following. My description of it is the result of attentive observation in a great number of cases that came under my care, and of much time and labour employed on the subject. I made minutes of every case as it occurred, though without any design, originally, of communicating them to the Public; but the long continuance of the epidemic having afforded me opportunities of observing it frequently, and of extending my remarks, I now venture venture to ſubmit to your conſideration the following account of this diſeaſe, to be inſerted, if you think proper, in the London Medical Journal.

Both ſexes were liable to this diſtemper, but eſpecially females; and it affected ſubjects of every age, excepting only ſuch as were far advanced in life; but it was more particularly frequent in children. I had occaſion to ſee ſeveral of theſe attacked with it, who were not a year old; and, in particular, one that had not reached its ſixth month, whoſe tonſils were covered with large white ſloughs, which continued ſeveral days, conſiderably affecting its ſwallowing and breathing; notwithſtanding which it recovered.

This diſeaſe conſtantly began with a ſoreneſs in the throat; but this ſoreneſs was generally ſo ſlight for the firſt twelve or twenty-four hours as to be taken but little notice of, the patients not complaining of pain in the throat, but only of ſome uneaſineſs when they attempted to ſwallow.

I was called to ſeveral children a day or two after the diſeaſe had taken place, and found them affected with ſo little uneaſineſs in the throat, or difficulty of ſwallowing, that their parents had not ſuſpected any complaint in the throat, although it was evident, on inſpection, from the ſtate of the tonſils, &c.

The throat, on being examined, appeared much inflamed, of a fiery red colour; and, in the courſe of the diſeaſe, the tonſils and uvula were, in ſome inſtances, very much ſwelled; but in others only moderately ſo. This appearance of the throat, however, differed exceedingly in different patients; and the ſwelling, even in caſes where it was conſiderable, was attended with but little pain or uneaſineſs, and that only when the patient attempted to ſwallow. This was more particularly obſervable during the ſummer months; for in the autumn and beginning of winter there was generally more ſwelling in the throat, attended ſometimes with conſtant pain, and frequently with very great difficulty in ſwallowing. In a few caſes the tonſils and uvula were ſo much ſwelled, when the diſeaſe was at its height, that it was with great pain and difficulty the patient could get down a ſpoonful of any fluid.

About the ſecond or third day ſloughs, which, in general, were of a whitiſh or yellowiſh colour, began to form on the tonſils, and ſometimes on the uvula, and ulceration took place. In many caſes deep ragged ulcers were formed on one or both tonfils; and in thefe cafes there was generally lefs pain and difficulty in fwallowing than where the parts were confiderably fwelled without ulceration.

When the throat had been much ulcerated, and large floughs formed, it was generally a long while before the latter were quite exfoliated or thrown off; and at the diftance of fix or eight days after all appearances of the difeafe had fubfided, and the patient, from his recovering his ftrength, fuppofed his throat to be quite well, I have, on infpection, found a white flough ftill remaining on one or both tonfils, which gradually feparated, and was thrown off.

The mucous glands were a good deal affected. About the third or fourth day a large quantity of mucus, which fometimes had a purulent appearance, was fpit up from the pofterior part of the fauces. The quantity that fome patients fpate up was aftonifhing, particularly for a day or two, towards the height of the difeafe.

There was likewife a confiderable difcharge of mucus from the noftrils, particularly in young children; and in thefe, in a few cafes, the eyes were fomewhat affected with the fame kind of difcharge.

The

The parotid and submaxillary glands were frequently enlarged, particularly in children, and sometimes suppuration took place. I met with one case, in a child of three years old, in which, when the disease was going off, an abscess formed on each side of the neck just under the lower jaw. The abscesses burst, and ill-looking ulcers were formed, discharging a very fœtid matter; a gangrene succeeded, and the disease proved fatal.

The tongue was generally covered with a very thick, moist crust, of a whitish or yellowish colour, which separated about the third or fourth day. When this crust had exfoliated, the tongue frequently appeared very red, as if raw, and was very tender, as was likewise the mouth, so that the patient could not bear any thing of a sharp taste.

In a few cases little exulcerations were observed about the tongue and mouth, which healed in a few days. When the tongue was clean early in the disease, (if the disease was violent) it afterwards became very dry and parched down the middle, the edges being moist. In a few patients the tongue, towards the decline of the disease, was covered with a brownish mucus. In general, the tongue becoming clean was not a ſign that the diſeaſe was diminiſhing.

The affection of the ſyſtem varied conſiderably. The inflammation in the throat, as I have obſerved, was the firſt appearance of the diſeaſe, occaſioning a ſlight ſoreneſs or ſtiffneſs in the parts*. In ſome this continued two or three days, and I have known it continue four or five, before the patient's general health was affected, though more frequently the ſyſtem became deranged before the end of twenty-four hours. Patients have ſometimes told me that the ſoreneſs in the throat came on nearly at the ſame time with the other ſymptoms; but, on

* This obſervation may ſeem to contradict what I have ſaid before (page 8), that the affection of the general ſyſtem firſt alarmed the friends of thoſe children in whom the diſeaſe had exiſted a day or two before the complaint in the throat was ſuſpected; but in thoſe caſes there was only a ſlight degree of ſwelling and tenſion of the tonſils, uvula, &c., ſo that the ſwallowing, at an early period of the diſeaſe, was but little affected, not ſufficiently ſo to attract the attention of the parents and byſtanders, who were not apprehenſive of any diſeaſe in the throat. This, however, we need not be ſurpriſed at, ſince in many, who were of an age to deſcribe their complaints, and in ſome inſtances even in adults, the uneaſineſs in the throat was ſo ſlight during the firſt day or two, that little attention was paid to it.

making making particular inquiry, I always found that the complaint in the throat had been coming on firft, and, in general, the day before the other fymptoms.

Sooner or later, however, after the difeafe had exifted in the throat, other fymptoms came on.

Sometimes a regular cold fit took place, fucceeded by the common fymptoms of fever, viz. increafed heat, frequent pulfe, thirft, &c.; but more commonly the patient felt frequent chillinefs through the day, and in the evening the ufual fymptoms of fever occurred which continued through the difeafe. Sometimes there was a regular exacerbation of the fymptoms in the evening, and a remiffion in the morning; at other times thefe febrile fymptoms continued without any obfervable increafe or diminution for feveral days, till the difeafe began to fubfide altogether.

In other patients no regular appearance of fever took place; but there was a flight derangement of the fyftem, as languor, lofs of appetite, and a pulfe rather more frequent than is natural; which fymptoms increafed in the evening, and, with the forenefs in the throat, occafioned reftlefs nights, fucceeded by fome remiffion in the morning.

Sometimes

Sometimes ficknefs and vomiting, and fometimes a diarrhœa, accompanied the other fymptoms; but the ftomach and bowels were feldom much affected at the beginning, and indeed not often at any period of the difeafe.

In many cafes a fcarlet eruption made its appearance on the fkin, (generally over the whole body) fometimes on the firft, though more commonly on the fecond or third day; but without appearing to give any relief to the other fymptoms.

In every inftance that came under my obfervation, where the difeafe terminated fatally, (excepting thofe who died of the dropfical fymptoms to be noticed hereafter) the fcarlet eruption took place in a very great degree. The fkin appeared very red, (which rednefs fome have compared with propriety to the colour of a boiled lobfter) dry, and contracted; and was intenfely hot to the touch: the pulfe at the fame time being frequent and contracted, but not fmall and weak, unlefs towards the clofe of the difeafe. There were fome exceptions, however, to this obfervation with regard to the pulfe. Where no fcarlet eruption appeared, the pulfe was often foft, fometimes weak; and in one or two cafes, which I met with in the fummer, and which which were attended with a great degree of debility, the pulſe was ſoft and very weak *.

The eruption had not always the ſame appearance. In general the ſkin was ſmooth, and the efflorescence did not riſe above its ſurface; but in ſome caſes there was a roughneſs plainly to be felt, particularly in one inſtance, in a child, where the eruption conſiſted chiefly of pimples, not much unlike the ſmall pox when it firſt makes its appearance.

The hands and feet, in many inſtances, were evidently ſwollen, and ſometimes the patient complained of an itching in the ſkin. When the eruption had once made its appearance, it generally continued with very little variation till the diſeaſe terminated; but where the diſeaſe was protracted to a great length, as, for inſtance, to the ninth or tenth day, the eruption loſt its brightneſs, and appeared rather of a darker colour.

* In adults, where there was but little appearance of fever, the pulſe generally beat about eighty or ninety ſtrokes in a minute; but where the febrile ſymptoms ran very high, it increaſed to an hundred and ten or an hundred and twenty ſtrokes; and under theſe circumſtances, in young ſubjects, it often exceeded this conſiderably.

In a few cafes, after the efflorefcence broke out, a number of little puftules made their appearance about the breaft, arms, and other parts of the body, of about the fize of millet feeds, and; upon being opened, were found to contain pus. Thefe, when firft formed, probably contained only a tranfparent fluid; but of this I am unable to fpeak with certainty, as I never happened to fee them till fome hours after they had appeared: they generally died away in twenty-four or thirty-fix hours. This was not a frequent appearance; yet in one family, where the mother and four children had the difeafe, thefe puftules appeared in all of them excepting one child.

In a patient, (a young man) to whom I was called in June, feveral white veficles appeared, about the fixth day, on different parts of the body, but particularly on the breaft and arms. Some of thefe were of the fize of a filver penny; others much larger: fome, when cut into, contained a fmall quantity of a tranfparent fluid; but others, upon being opened, appeared quite dry. By the next day they all dried away, and the fkin covering them peeled off.

In November it occurred to me to fee another cafe, in which veficles of a different kind appeared peared on the ſkin. In this patient, who was likewiſe a young man of a robuſt conſtitution, on the ſixth day ſeveral veſicles were formed on the arms, thighs, and legs. Some of theſe were as large as a half crown, and, if not accidentally broken, became much diſtended, partly with a yellow ſerous fluid, and partly with a gelatinous ſubſtance of the ſame colour. They were ſurrounded with a good deal of eryſipelatous inflammation, and were not followed by a diminution of the other ſymptoms; but, on the contrary, from the pain and uneaſineſs which they occaſioned, they made the patient more reſtleſs, and rather aggravated the diſeaſe. In this caſe the diſeaſe diminiſhed on the ninth day; the urine, which before had contained only a light cloud, now perfectly ſeparated, and depoſited a copious farinaceous ſediment. The frequency of the pulſe, and other ſymptoms, ſubſided conſiderably; indeed the heat (which was never ſo intenſe in this patient as in many others) had been gradually abating for two or three days, although none of the other ſymptoms were leſſened before this day*.

* In this patient apthæ appeared as the diſeaſe was going off, and continued two or three days after the patient was getting better.

The scarlet eruption was by no means a constant appearance, and therefore not an essential character of this disease.

The degree in which the system was affected was not always in proportion to the degree of violence of the disease in the throat.

As this disease differed much in the degree of violence in different patients, so did the time of its duration vary. Where the affection of the throat was slight, and attended with little affection of the system, the symptoms subsided in a few days; in general upon the fifth or sixth day, sometimes later, the symptoms diminished.

In some cases critical appearances took place on the sixth day; such as a diminution of the swelling in the throat, and difficulty of swallowing where that had been troublesome; a diminution of the heat of the body, and of the frequency of the pulse; a moisture on the skin, and sometimes a change in the appearance of the urine. More frequently, however, the disease diminished gradually, without any appearance of crisis.

With regard to the appearance of the urine, it may be observed, that it frequently afforded but little information of the state of the disease, or of the changes that were likely to take place in the patient. Sometimes the urine varied in its appearance at different periods of the difeafe in the fame patient. I have likewife feen it turbid while the difeafe continued; and when the fymptoms began to fubfide, the urine, in fome cafes, appeared clear, without a fediment; while, in others, a perfect feparation took place with a copious fediment. On the other hand, I have obferved it to be clear while the difeafe continued, and become afterwards turbid. Laftly, I have feen it of a pale colour, containing only a light cloud throughout the difeafe, and continuing with the fame appearances after the difeafe went off.

Delirium feldom took place; but a dull pain in the head was not an unufual fymptom in adults.

The difeafe was attended with but little depreffion of ftrength, excepting in a few cafes I met with in the fummer; but I never faw any figns of putrefaction.

In every cafe where the fcarlet eruption appeared, as foon as the difeafe went off the fkin began to fcale, and a complete defquamation took place; but this I did not obferve to be the cafe where there was no efflorefcence on the fkin. Sometimes this change began to take place before the difeafe was entirely gone off. In a girl, about feven years old, the fkin feemed to be falling off in branny fcales two or three days before fhe died. Where the fcarlet eruption prevailed very much, the fkin, in moft inftances, (I think I may fay conftantly) was remarkably dry.

The difeafe was certainly infectious, as appeared from its attacking feveral in a family, and fome nurfes who attended the fick.

That the inflammation in the throat is to be confidered as the effential character of the difeafe is evident from this circumftance, that many had the difeafe in the throat in whom no fcarlet efflorefcence appeared on the fkin; but I did not meet with a fingle cafe where this appearance took place in the fkin without fome affection of the throat.

The firft inftance of the difeafe that occurred to me I fuppofed to be a cafe of fcarlet fever fimply. The patient was a child, three years old, who was feized, on April the 13th, with fymptoms of fever, which continued the whole night, and in the morning an univerfal fcarlet eruption was obferved. As the child was not obferved to fwallow with difficulty, and no inftance of fore throat had then occurred, I confefs it did not ftrike me as a cafe of that kind, and therefore I did not think of examining the throat. The tongue was covered with a thick white cruft, exactly fimilar to what I have met with in almoft every cafe fince; and in four or five days the fubmaxillary and parotid glands were fwelled confiderably. The child fwallowed then with difficulty; a large quantity of mucus was difcharged from the nofe, and other appearances fucceeded, fimilar to what I have met with in a variety of cafes fince, fo that I have not a doubt of its having been the fame difeafe.

This difeafe carried off but few patients, confidering the great number who were affected with it. Thofe, whom I had an opportunity of feeing, who unhappily fell victims to its violence, continued feveral days from its attack. One or two children lived to the ninth or tenth day; but in fome inftances the difeafe proved fatal at an earlier period, and in one or two even fo foon as the third day. I met with one fingular inftance where the patient lived nearly a month from the firft attack. In this cafe, at the time the difeafe was expected to fubfide, there was but little abatement in its appearance, and the patient continued to live, under an irregular train of ſymptoms of great irritability, to the twenty-ſeventh day. In this patient apthæ appeared at the end of the ſecond week. Caſes ſomewhat ſimilar to this are mentioned by Dr. Withering in his Treatiſe on the Sore Throat and Scarlet Fever *.

In this diſeaſe the difficulty of ſwallowing did not appear to ariſe ſo much from the uneaſineſs occaſioned by ſubſtances paſſing over the diſeaſed part, as from the difficulty in bringing the neceſſary muſcles into action.

There was ſomething peculiar in the appearance of the throat; for large ſloughs were often formed, and when they were ſeparated (which I have obſerved was frequently not till ſome days after the diſeaſe had ſubſided) the ſurface of the part had not the appearance of a common ulcer, with granulating fleſh ſecreting pus, but ſeemed to be ſkinned over: I ſay frequently, for this was not conſtantly the caſe. The ſlough often looked as if it was nothing more than the coagulable lymph thrown out on the ſurface of the tonſils, &c., and coagulated, and this I believe was ſometimes the caſe; but on viewing the parts, even with a ſlight degree of atten-

* Page 8.

tion, in many cafes there appeared evidently a lofs of fubftance, and fome appearance of pus, which muft have arifen from ulceration.

As this difeafe affumed fuch very different appearances as thofe I have been defcribing, it may be fufpected perhaps that there were two difeafes prevailing at the fame time; one properly called fcarlatina anginofa, and another, a different difeafe, verging to what is called the true ulcerous or malignant fore throat. But this I am confident was not the cafe; for the fame contagion acting on different conftitutions occafioned great variety in the appearance of the difeafe; fo that fometimes a patient, in whom the fcarlet appearance was very confiderable, communicated the difeafe to another, in whom no fuch appearance took place, and *vice verfa*.

This feemed to be the fame difeafe as that which prevailed at Birmingham in the year 1778, and which is defcribed by Dr. Withering; or at leaft a modification of it, for in feveral circumftances there was a material difference, viz. in the appearance of the throat, ftate of the pulfe, degree of debility, &c., as muft be evident to any one who will compare the defcriptions of the two difeafes.

This diſeaſe is called by many ſcarlatina anginoſa. It may appear trifling to diſpute about names; yet when names are applied, which give an imperfect or wrong idea of a diſeaſe, they are certainly exceptionable: and with the utmoſt deference to thoſe reſpectable authors who have deſcribed this diſeaſe under the name of ſcarlatina anginoſa, I cannot help thinking it an improper term, as it gives an idea of the ſcarlet eruption being an eſſential part of the diſeaſe, and conſtantly making its appearance, which is not the caſe; for I have met with many inſtances, as I have already mentioned, where the diſeaſe exiſted, and that in a great degree, without any diſcolouration of the ſkin. Scarlatina will, therefore, only apply to the diſeaſe under certain circumſtances.

Sydenham has deſcribed a ſimple ſcarlet fever where there was no affection of the throat. It is deſcribed alſo by other authors; yet it is a diſeaſe which rarely occurs: for Dr. Cullen obſerves, that in all the inſtances of ſcarlet fever which he has ſeen, the diſeaſe, in almoſt all the perſons affected, was attended with an ulcerous ſore throat, or was what Sauvages calls ſcarlatina anginoſa; yet Dr. Cullen ſeems fully of opinion opinion that there is a ſcarlet fever not neceſſarily connected with an affection of the throat*.

When I firſt met with this diſeaſe, ſeeing large ſloughs quickly formed on the tonſils, I fully expected that it would ſoon aſſume a putrid appearance, particularly as the ſummer was advancing, and that it muſt be conſidered as the true ulcerous ſore throat. I was the more inclined to this opinion, as I remembered that the diſeaſe, called by ſome authors ſcarlatina anginoſa, was conſidered by the learned and ingenious Dr. Fordyce, in his lectures, as being the ſame diſeaſe as that which he calls the eryſipelatous ſore throat, or what is commonly called the ulcerous or malignant ſore throat. But our epidemic not being attended with that depreſſion of ſtrength, irritability, and diſpoſition to aſſume a putrid appearance, which ſo ſtrongly characterize the eryſipelatous or ulcerated ſore throat, ſoon convinced me that it was a different diſeaſe, or ſo different a modification of that diſeaſe as to require a different mode of treatment; for in the eryſipelatous ſore throat the moſt ſucceſsful practice (and that which is

* See Firſt Lines of the Practice of Phyſic, 4th Edition, Vol. II. page 189.

particularly inculcated by Dr. Fordyce in his lectures) is to give large quantities of Peruvian bark, and that early in the disease, otherwise the patient is in danger of being cut off very soon: but I met with very few cases where the Peruvian bark was necessary or proper to be given, excepting towards the decline of the disease, and then it was but seldom required, and in moderate quantity. In by far the greater number of instances relaxants alone were depended upon throughout the whole course of the disease; which in the true erysipelatous sore throat would have been a very unsuccessful practice.

This disease differed from the common inflammatory angina in having sloughs, and oftentimes ulcers, formed on the tonsils, &c.; and in there being no disposition to terminate in suppuration, not even in those cases where there was considerable inflammation and painful swelling, without any or with very little appearance of slough or ulcer*. Moreover, the state of the system was not general inflammation,

* I met with one case of the common inflammatory angina in June, which, being left to itself, suppurated; and was attended with the usual symptoms of that disease.

commonly called inflammatory fever, which confifts in an increafed and ftrong action of the blood veffels, as is the cafe in the pure inflammatory angina.

In this, as in every other inftance, we are not to diftinguifh the difeafe from other difeafes by one or two fymptoms, but by a comparative view of the whole difeafe. With regard to the method of diftinguifhing difeafes in general, I cannot help mentioning an obfervation of Dr. Fordyce. — "Difeafes," fays this experienced and fagacious Phyfician, "have been "attempted to be diftinguifhed from one ano-"ther by pathognomonic fymptoms, that is, "appearances which were always prefent when "the difeafe was prefent, and abfent when the "difeafe was abfent; and lately they have been "attempted to be diftinguifhed in another man-"ner, in that fame kind of divifion which has "been introduced to diftinguifh plants, by ma-"king artificial claffes of them, joining toge-"ther difeafes which only agree in one fymp-"tom, as pain in the fide for example, &c., "but with as little fuccefs. If there were pa-"thognomonic fymptoms of difeafes, that we "could make that kind of claffification, and "fo diftinguifh them, it would, no doubt, be

"very advantageous; but the ſame diſeaſe varies ſo much in different caſes, that neither of "theſe things can be done: we can only diſtinguiſh them by being maſter of the whole hiſtory of the diſeaſe."

*Dropſical Symptoms.*

After the diſeaſe had gone through the courſe juſt now deſcribed, a ſecond ſet of ſymptoms frequently aroſe, manifeſting an anaſarcous ſtate of the whole body, which to many were troubleſome, and to ſome even fatal, although they came on in ſo gentle a manner as too frequently to give the patient little reaſon to ſuſpect any danger from them. Theſe ſymptoms generally took place about a week or two after the other morbid appearances had ſubſided. The patient, whoſe conſtitution had not yet recovered its ſtrength, became univerſally anaſarcous; the breathing became ſomewhat laborious, particularly when in an horizontal poſture; the urine was ſmall in quantity; the appetite, which had in ſome meaſure returned, now diminiſhed, and debility of the whole conſtitution ſlowly increaſed.

This part of the complaint, in ſome, was readily removed; but in others it was very obſtinate

ſtinate and alarming. I met with two caſes where it proved fatal, and that in a very ſhort time. The firſt of theſe caſes occurred to me in May. The patient was a girl, between three and four years old, who, about a week after the other ſymptoms had ſubſided, was obſerved by her mother to be ſomewhat ſwelled univerſally. The next day the ſwelling rather increaſed, and the child breathed with ſome difficulty; but as there were no other threatening ſymptoms, and the patient's general health did not ſeem much impaired, her mother thought it a matter of little conſequence, and ſuppoſed that by ſome purgative medicine the ſwelling might be carried off. Accordingly the next morning (which was the third from the coming on of the anaſarca) ſhe gave the child a doſe of jalap, which purged her moderately. In the evening, however, the patient was evidently getting worſe, though incapable, from her tender age, of giving a particular account of her complaints; but laying her hand on her ſtomach, ſhe expreſſed a ſenſe of pain in that part. She breathed with difficulty; her ſkin felt very hot, and ſhe paſſed a very reſtleſs night. The following morning the difficulty of breathing increaſed very faſt; ſhe became inſenſible; her countecountenance very pale, and much altered; and in two or three hours ſhe died. I did not ſee this child till within an hour of her death. I requeſted leave to open the body, but the father of the child was unwilling.

The other inſtance was that of a boy, four years old, in whom the diſeaſe began in the ſame way, but its progreſs was not ſo rapid. When the anaſarcous ſymptoms commenced, the parents of the child, as in the other caſe, were not apprehenſive of any bad conſequence, no ſymptoms, which to them were alarming, having appeared till a day before the child died. The diſeaſe in the throat, and the ſymptoms immediately connected with it, had gone off about ten or twelve days when the child was obſerved to be univerſally ſwelled. His countenance, at the ſame time, was pale; his appetite failed; he made but a ſmall quantity of urine; and, in an horizontal poſture, his breathing was much affected, ſo that he paſſed very reſtleſs nights. On the 28th of September, which was nearly a fortnight after theſe dropſical ſymptoms had been firſt obſerved, the ſymptoms increaſed conſiderably, particularly the affection of the breath; and the day following I was deſired to ſee him for the firſt time. He appeared to be really *in*

*articulo*

*articulo mortis*, his breathing being very laborious, his face bloated, his eyes dim, his countenance of a dead palenefs, and his lips of a dark purple hue. He foamed at the mouth; his pulfe was fmall, frequent, and irregular; every mufcle of the body appeared to be in a ftate of convulfive agitation: he feemed to be totally infenfible, and was incapable of fwallowing. Thefe fymptoms, to my furprife, after a few hours, diminifhed, and he appeared to be much altered for the better; but in the evening they returned again with great violence, and in a few hours he died.

A girl, about ten years old, was likewife attacked, in October, with very threatening and alarming fymptoms. In two days after the dropfical fwelling had commenced, very great difficulty of breathing came on, which appeared evidently to be a fpafmodic affection. It continued feveral hours; but by means of repeated dofes of æther her breathing became eafier and calmer.

This affection of the breathing returned with confiderable violence, at intervals, for ten or twelve days, fometimes three or four times in the courfe of a day and night, and was as often removed by a dofe of æther, which was always kept kept in the room to be given immediately on the coming on of this complaint. Moreover, ſhe was ſubject to ſuch violent retching for more than a week, that ſhe could hardly keep any thing on her ſtomach, either of food or medicine, excepting the æther. Her countenance was pale and bloated; there was ſome ſwelling all over the body, though not in a great degree; and ſhe was ſo debilitated, that ſhe could not ſit up many minutes together. At length, however, this ſpaſmodic affection returned with leſs frequency and violence, and her ſtomach became more ſettled, ſo that ſhe could bear ſome ſtrengthening medicines, viz. extract. caſcarill. and myrrh, in the form of pills, in ſmall doſes. Afterwards ſhe took larger doſes of theſe medicines, was able to eat a little nouriſhing food, and ſeemed to gain ſtrength. In the mean while ſhe made a larger quantity of urine, and the ſwelling gradually ſubſided. The ſame mode of treatment was continued for ſome time, with the addition of the *ferrum ammoniacale*; her ſickneſs entirely left her, her breathing became eaſy and natural, and ſhe recovered her former health.

Of

### *Of the Mode of Treatment.*

At the beginning of the difeafe, if the ftomach was affected with ficknefs, an emetic was ufeful; and in moft inftances I thought it beft to begin with a remedy of this fort, not only with a view to clear the ftomach of its contents, but likewife to promote the fecretions, in general, by producing univerfal relaxation in the fmall veffels. Afterwards, if the body was coftive, rhubarb, manna, fenna, &c., given fo as to procure a few ftools, were ufeful; but violent purging was to be avoided, as it tended to induce too great a degree of debility without diminifhing the difeafe. If the bowels were in the oppofite ftate, (viz. if there was a purging) after an emetic, a dofe of rhubarb was proper; and if the purging ftill continued, the *pulvis e creta compofitus*, and ipecacuanha in fmall dofes, were given with advantage, or fmall quantities of opium. This laft medicine, however, unlefs the purging was very violent, was not attended with much advantage; and indeed, in general, it was rather hurtful in every ftage of the difeafe. I tried it towards the clofe in cafes where the difeafe had been protracted to a confiderable

 length, length, but it was almoſt conſtantly diſpoſed to act too powerfully as a narcotic; and after this effect was over, the irritability of the body was evidently much increaſed: I likewiſe adminiſtered it in different doſes, but its effects were ſuch, that I found it neceſſary to omit it almoſt entirely.

Bleeding, from the ſyſtem, was, in general, not indicated. In one inſtance only, which occurred to me in April, I ventured upon it. The patient was a ſtrong young man, who had conſiderable inflammation and ſwelling in the throat, and pain in the head, with heavineſs; his pulſe was full and ſtrong. The blood was not ſizy; and although the patient recovered, the bleeding was not attended with ſuch advantage as to induce me to adopt the ſame practice in any other caſe. But topical blood-letting, viz. by applying leeches to the temples, was uſeful in the few inſtances in which I tried it, where the patient felt much pain in the head, with giddineſs; and in one or two other caſes where delirium took place, it was likewiſe attended with advantage.

The frequency and contraction of the pulſe, and very great dryneſs of the ſkin, with exceſſive heat, which were ſuch conſtant ſymptoms in in cases where the eruption appeared, and likewise in many instances where there was no eruption, strongly indicated the use of relaxants. Accordingly they were freely administered, viz. the preparations of antimony or ipecacuanha, and the neutral salts.

The antimonial which I generally used was the *antimonium tartarisatum*, given in as large doses as the stomach would bear without sickness: I often joined with it some neutral salt, viz. *aqua ammoniæ acetatæ*, or *kali acetatum*; or, if a laxative was wanted, I gave *kali tartarisatum*, or *kali vitriolatum*. In cases where the *antimonium tartarisatum* acted on the intestines too much, or if there was already a disposition to purging, I gave ipecacuanha, in doses of about a grain, every four or five hours.

The effect of these medicines was not, in general, very striking, though I am persuaded that, when they were regularly persisted in, they diminished the violence of the disease by reducing the frequency of the pulse and intense heat; and although their relaxing effects were not shewn by a moisture on the skin, which is commonly the case in other diseases, they were nevertheless advantageous. From the perfect dryness of the skin, which I constantly observed

 when when the ſcarlet eruption made its appearance in a great degree, and the deſquamation of the ſkin, which never failed to take place, I ſuſpect that the organization of the cuticle was ſo far altered by the peculiar nature of the diſeaſe as to render it almoſt impoſſible for any ſweat to break out.

I was the more convinced of the propriety of perſiſting in the uſe of relaxants, from obſerving that whenever a gentle ſweat took place ſpontaneouſly, the diſeaſe terminated ſooner, and was leſs violent. In ſome caſes where there was an obſtinate dryneſs and heat of the ſkin, I applied fomentations to the lower extremities for a conſiderable time together, and repeatedly, but I never ſaw any advantage from them.

If, towards the decline of the diſeaſe, the pulſe became weak, and the heat of the ſyſtem diminiſhed, and the patient's ſtrength ſeemed to be ſinking, it became neceſſary to give ſtimulants, and to allow them wine. Indeed, at an early period of the diſeaſe, this mode of treatment was often uſeful where there was no great heat, (which was often the caſe where no eruption appeared) and the pulſe was rather ſoft. If there was at the ſame time contraction and dryneſs of the ſkin, ſmall doſes of *antimonium*

*tartarisatum*, or of ipecacuanha, were alſo given with advantage. The medicine which I commonly uſed as a ſtimulant was the contrayerva root, given in ſubſtance, and ſometimes joined with camphor. In a few caſes which I met with in the ſummer months, and which were attended with an unuſual degree of debility, the Peruvian bark was given in large doſes with advantage; but in a few other inſtances in which I employed this medicine, with a view to ſupport the patient's ſtrength, it was not adminiſtered till towards the decline of the diſeaſe, and then only in decoction, in which form it was attended with good effects.

In ſeveral caſes where there was a great deal of inflammation in the throat, with pain and ſwelling, I applied a bliſter to the throat, but without ever being able to obſerve any advantage from it. I therefore generally uſed a ſtrong volatile liniment, viz. *linimentum ammoniæ fortius*, and, if there was much external ſwelling, it was of ſome uſe, otherwiſe I could not ſee any benefit ariſing from it. What I found moſt uſeful, as an external application, was the *linimentum camphoræ*. This I did not try till towards the cloſe of the epidemic, and therefore had not an opportunity of ſeeing it applied in a great great number of inftances; but where it was ufed it certainly produced good effects. This liniment ftimulated the fkin, and by a pretty conftant application excoriated it. I found it better, therefore, not to apply it too frequently: a piece of lint was dipped in it and laid on the throat, and renewed every four, five, or fix hours, or oftener if the difeafe had run to a confiderable height before it was ufed, and the fwallowing was become very painful. The patient was enabled to fwallow much better after its application, probably from its ftimulus affecting the mufcles fubfervient to deglutition.

It was neceffary to gargle the throat frequently, as a large quantity of mucus was apt to be collected about the tonfils, &c. The common gargles were ufed for this purpofe, viz. *infufum rofæ*, with the *mel rofæ* or *tinctura myrrhæ*; or a decoction of contrayerva root, &c. In cafes where the patient complained much of pain in the head, a blifter on the back was ufeful.

Many inftances of this difeafe occurred, in children, in which nature might be faid to effect a cure with little or no affiftance from medicine, topical applications excepted; for in thefe cafes the quantity of internal remedies we were able to get down was fo fmall, and given after fo long long intervals, that the diſeaſe could hardly be ſuppoſed to be ſubdued by ſuch treatment.

The diet conſiſted of food of eaſy digeſtion, as panada, ſago, gruel, barley water, or milk and water, &c., and where there was much debility wine (as I have already mentioned) was allowed. In ſlighter caſes, where the ſyſtem was but little deranged, and the ſtomach not much affected, more freedom in diet was allowed, and animal broths were taken without inconvenience.

In the dropſical ſtate the treatment was very ſimple, and I believe if the remedies had been early applied, they would have been generally ſucceſsful. The indications of cure were to reſtore the patient's ſtrength, and to increaſe the urinary ſecretion, in order that the preternatural accumulation of water in the cellular membrane might be abſorbed and carried off. Accordingly ſtrengthening medicines and diuretics, ſuch as *rad. colombæ*, *flor. chamæmeli*, &c., with the fixed alkalis, were adminiſtered with advantage; and at the ſame time a nutritious diet was recommended to the patients.

*Cheſham*,

December 14, 1788.

